# Oral health service utilization patterns among preschool children in Beijing, China

**DOI:** 10.1186/s12903-018-0494-6

**Published:** 2018-03-06

**Authors:** Mengru Xu, Chao Yuan, Xiangyu Sun, Menglin Cheng, Yanyi Xie, Yan Si

**Affiliations:** Department of Preventive Dentistry, Peking University School and Hospital of Stomatology, 22 South Zhongguancun Avenue Haidian District, Beijing, 100081 China

**Keywords:** Oral health service utilization, Preschool children, China

## Abstract

**Background:**

The utilization of oral health services in children remains at a relatively low level in China. However, little is known about the utilization patterns and related factors. The objective of this study was to explore the patterns of oral health service utilization and to determine the related factors among preschool children in Beijing, China, based on the Andersen behavioral model.

**Methods:**

A cross-sectional study of 1425 preschool children aged 2 to 6 years was carried out in five kindergartens in Beijing, China. A questionnaire investigation of parents/caregivers was performed to collect information on oral health service utilization. Oral health needs were evaluated through oral health examinations. Chi-square tests, t-tests, multivariate logistic regression and negative binomial regression were used in this study to identify the variables associated with oral health service utilization.

**Results:**

In total, 648 (45.5%) children had utilized oral health services in the past 12 months, while 24.3% had utilized preventive oral health services. Routine checkups and receiving preventive measures accounted for 63.2% of the children who utilized oral health services in the past 12 months. Children were more likely to have utilized oral health services in the past 12 months if they attended kindergartens with regular oral health care resources, if their oral health status as perceived by their parents/parents was “fair” or “poor”, if they had more decayed, missing and filled teeth (dmft) and if they had experienced more dental pain. In addition, children with more dental pain and more access to oral health services, whose oral health status was perceived by parents/caregivers as worse tended to have utilized oral health services more frequently in the past 12 months.

**Conclusions:**

In conclusion, we found a strong association between access to regular oral health care resources and oral health service utilization among preschool children. Children whose oral health status was perceived by parents/caregivers as worse and who had more dental pain were more likely to have utilized oral health services in China.

**Electronic supplementary material:**

The online version of this article (10.1186/s12903-018-0494-6) contains supplementary material, which is available to authorized users.

## Background

Oral health is a critical component of general health, and the poor oral health status of children in China is still a significant public issue [1–3]. At present, the utilization of oral health services in children remains at a relatively low level in China [1–8]. However, little is known about the utilization patterns and related factors.

It is recommended that children visit a dentist within 6 months of the eruption of their first tooth [9]. One previous study based on the American population showed that once a child went to the first dental visit, 94% would continue with regular checkups [10]. Furthermore, regular dental visits in childhood support oral health service utilization into adulthood and can provide opportunities for preventive interventions and oral health education for the children and their parents or caregivers. However, disparities and inequalities of oral health service utilization in children have been found in most developed and developing countries [11]. A study in 11 European countries suggested that a considerable proportion of inequalities in dental care use was established in childhood and persisted throughout an individual’s life-course [12]. There are differences in the utilization of oral health services between children in Eastern, Central and Western China, as well as between children in urban and rural areas, according to the 3rd National Oral Health Survey in 2005 [13]. However, these disparities in China were not investigated in depth, as little evidence was available. To achieve the goal of “Healthy China 2030”, it has been recognized by the government that more attention should be paid to oral health in children and inequalities in the utilization of health care services [14].

Studies concentrated on the utilization of oral health services among preschool children have been mostly carried out in developed countries [15–18] and in a few developing countries, such as Brazil, Mexico and Thailand [19–22]. China, which is one of the largest developing countries in the world, has an enormous preschool population, and 77.3% of 5-year-old Chinese children have never visited a dentist [13].

Before school admission, parents or caregivers play an important role in their children’s utilization of oral health services. There is considerable evidence from previous studies that children’s utilization of oral health services may be influenced by parental education level [18, 20, 23], socioeconomic status [16, 19, 20, 24, 25], oral health beliefs [25–27] and their perception of their children’s oral health status [24, 25, 28–30]. Other demographic characteristics of the children, such as age [15, 20, 21, 23, 25] and gender [18, 28] have also been found to be related to oral health service utilization in some studies. Dental insurance [15, 23] and dental fear [22] also affect oral health service utilization in children. All these factors can be included in the Andersen behavioral model [31], which describes the interrelationships between population characteristics (predisposing factors, enabling resources and need, PEN), health behaviors and health outcomes. This model has been widely used to investigate variables that explain health service use and ways to improve it and has been applied in other research using parents/caregiver-reported information to examine children’s oral health service utilization [28, 32].

A systematic review based on surveys collected from 1987 to 2013 in mainland China showed a declining trend of early childhood caries (ECC) but there is still a high level of prevalence (77.9% in 1987–1994 to 56.4% in 2010–2013) [1]. A slight increase in the teeth that were treated (3.1% in 2005–2009 to 4.9% in 2010–2013) was reported [1], which implied an unmet oral health care need among children. In China, previous research mainly concentrated on the utilization of oral health services among adults [33, 34], and data for children, especially preschool children, were only found in a few regional studies, most of which were concerned with oral health behaviors but were not specific to oral health service utilization [3–8]. These regional studies have shown that 12.3%~ 42.3% children had accessed oral health services within the past 12 months. However, little was known about the factors related to pediatric oral health service utilization in China. Thus, identifying the patterns and determinants of oral health service utilization among preschool children will assist in the creation of health policies and the improvement of public oral health in China.

The objective of this study was to explore the patterns of oral health service utilization and to determine the factors associated with oral health service utilization among preschool children in Beijing, China, based on the Andersen behavioral model.

## Methods

### Study population

This was a cross-sectional study among 1425 preschool children (2–6 years old) in Beijing, China. Multistage randomized cluster sampling was used in our study. First, we chose the Chaoyang and Haidian Districts the population of which could be representative of that of Beijing, as they both have urban and rural areas [35]. Second, we used randomized cluster sampling to select five kindergartens in these two districts. Three of the five kindergartens cooperated with a professional dental hospital, which could provide children with regular oral health care such as routine checkups and treatments.

All parents of enrolled children signed informed consent. This study protocol was approved by the Human Research Ethics Board of Peking University Health Science Center, Beijing (No. IRB0000105210090).

### Data collection and measures

Self-reported questionnaires were finished by parents or caregivers. The questionnaire used in the present study was developed from the questionnaire of the 3rd National Oral Health Survey of China in 2005 [13].

The outcome variable of our study was oral health service utilization (whether services were used and at what frequency) in the 12 months prior to data collection. Furthermore, the reason for the latest dental visit in past 12 months was recorded for children who had utilized oral health services.

Demographic variables (i.e., age and gender of children), socioeconomic variables (i.e., highest degree of education of parents, occupation of parents and annual household income), health belief variables, dental pain experience of children in the past 12 months and parents’ perceptions of the oral health status of their children were also obtained from the questionnaires. To evaluate parental oral health beliefs, we asked four questions about their knowledge and four questions about their attitudes towards children’s oral health (see Additional files 1 and 2). For the knowledge-related questions, a score of 1 was given for each correct answer, and a score of 0 was given for an incorrect answer or for an answer of “I don’t know”. To evaluate the attitudes of parents/caregivers towards oral health, there were three possible responses to each statement: “agree”, “disagree” or “do not care”. For each question, caregivers showing a positive attitude received a score of 1. The total scores for knowledge and attitude were calculated by summing the scores of all eight questions. The final scores ranged from 0 to 8, which determined the oral health knowledge and attitude scores. For analysis, we categorized the scores into two groups “score ≥ 5” and “score < 5”. We used three scaled categories to measure parental perceptions of their children’s oral health status, which were “excellent/good, fair, poor”.

Oral health care needs were estimated by the results of oral health examination. All subjects were examined by one of two examiners to identify dental caries using a flat dental mirror, a Community Periodontal Index (CPI) probe and natural light. For the detection of caries, we used the methods and criteria recommended by the World Health Organization (WHO) [36]. After the oral health examination, we informed the parents or caregivers of the children’s oral health status and advised them to seek professional treatment if needed. Through the standard consistency examination, the Kappa values for examiners were calculated. The inter-examiner Kappa value before the survey ranged from 0.82 to 0.91. During the survey, a standard assessment of consistency was performed, where the inter-examiner Kappa value ranged from 0.80 to 0.93, and the intra-examiner value ranged from 0.82 to 0.97.

### Conceptual model

This study is based on the health behavioral model proposed by Andersen et al. [31], which asserts that care-seeking behavior is determined by predisposing, enabling, and need (PEN) factors. Predisposing factors, including demographic characteristics (“age” and “gender” of the child, “education level” and “occupation” of the parents) and health beliefs (parental “oral health knowledge and attitude”), are those that exist prior to disease and can be either mutable or immutable. Enabling factors, such as socioeconomic factors (“household income”, “regular resource of oral health care”), include resources that affect an individual’s ability to access the health care system. Need factors (“parents’ perceptions of the oral health status of their children”, “dental pain experiences”, “dmft value”) indicate an illness that requires the use of services and include both perceived and evaluated health status.

### Statistical analyses

First, descriptive statistics and bivariate associations between each variable of interest and the outcome variable were analyzed. A series of Chi-square tests and t-tests were carried out to determine which independent variables showed a statistically significant association (*P* < 0.05) with the outcome variable. Second, a logistic regression model was conducted to determine the factors associated with oral health service utilization using odds ratios (OR) and 95% confidence intervals (CI). The negative binomial regression (NBR) model using the incidence rate ratio (IRR) and 95% confidence intervals were used to examine associations between the frequency of oral health service utilization and the independent variables. The NBR, when compared with the Poisson regression, was the most appropriate model for the outcome variables due to over-dispersion in the number of dental visits (likelihood ratio test of alpha = 0: LR = 695.58, *P* < 0.001). We used multicollinearity diagnostics to determine that there was no multicollinearity between independent variables (variance inflation factor, VIF = 1.00~ 1.98, mean VIF = 1.32). These analyses were conducted using STATA statistical software (SE version 14.1, Stata Corp, College Station, TX, USA).

## Results

### Descriptive characteristics

All the 1631 selected children received oral health examinations, while 1425 parents or caregivers finished the questionnaires (with a response rate of 87.4%). Most of the respondents were children’s parents (mothers: 73.9% and fathers: 18.5%), while others were caregivers, such as grandparents or other relatives. In total, 648 children (45.5%) were reported to have used oral health services in the past 12 months, and 347 children (24.3%) were reported to have used dental preventive measures in the past 12 months. The mean number of times oral health services had been used in the past 12 months was 1.00 ± 1.86.

Table 1 demonstrates the descriptive characteristics of the sample. Half of the population was male, and the mean age of the children was 4.08 ± 1.13 years. The prevalence of caries in this population was 54.5%, and the mean dmft value was 2.14 ± 2.95. Most of the parents or caregivers (73.5%) had oral health knowledge and attitude scores higher than 5. More than half of the families had an annual household income beyond the level of 100,000 Chinese Yuan (CNY), which was the average income of all Beijing households in 2011 [35]. One-third of the parents or caregivers only received high-school education or less, while a quarter of them had received education higher than a Master’s degree. More than one half of the parents or caregivers (59.5%) had administrative or professional occupations. Nearly 70% of the children attended a kindergarten with regular resources for oral health care. There were 10.5% of the parents or caregivers who perceived their children’s oral health status as “poor”, whereas nearly one-fifth of them reported dental pain experiences of their children in the past 12 months.

**Table 1:** Descriptive characteristics of the sample of preschool children aged 2–6 years in Beijing, China (*n* = 1425)

Variables	Numbers	Percentage
Predisposing factors		
Gender		
Male	735	51.6%
Female	690	48.4%
Age		
≤3 years old	506	35.5%
> 3 years old	919	64.5%
Occupation of parent or caregiver		
Administrator/Professional	848	59.5%
Other	577	40.5%
Final education degree of the parent or caregiver		
High school or lower	460	32.3%
University or college	591	41.5%
Master’s degree or higher	374	26.3%
Parental oral health knowledge and attitude score		
< 5	663	46.5%
≥ 5	762	73.5%
Enabling factors		
Annual household income		
≤100,000 CNY	683	47.9%
> 100,000 CNY	742	52.1%
Regular resource of oral health care		
Yes	994	69.7%
No	431	30.3%
Need factors		
Parental perceived oral health status of the child		
Excellent/good	882	61.9%
Fair	393	27.6%
Poor	150	10.5%
Parental report of dental pain in the past 12 months		
Yes	336	23.6%
No	1015	71.2%
Unknown	74	5.2%
Dmft value		
dmft = 0	649	45.5%
dmft > 0	776	54.5%

Among the 648 children who used oral health services in the past 12 months, 579 parents/caregivers reported the reason for the latest dental visit; 38.3% went to a dentist for routine checkups, and 21.6% used the services for preventive measures, while only one-tenth went to a dentist because of dental trauma or toothache (Table 2).

**Table 2:** Reasons for the latest dental visit in the past 12 months (*n* = 579)

Reasons for the latest dental care	Numbers	Percentage
Emergency for trauma	11	1.9%
Acute toothache	29	5.0%
Chronic toothache	39	6.7%
Examinations for oral problems	144	24.9%
Routine check-up	222	38.3%
Application of preventive measures	125	21.6%
Aesthetic needs	9	1.6%

### Factors associated with oral health service utilization in the past 12 months

Table 3 shows several significant differences between the groups of children who had and had not utilized oral health services in the past 12 months. Children who had dental pain in the past 12 months, whose oral health status was poor, whose parents or caregivers had a higher education level, or whose parents worked as administrators or professionals were more likely to have used oral health services in the past 12 months. Parents or caregivers who had oral health knowledge and attitude scores ≥5 seemed more likely to take their children to a dentist compared to parents with scores < 5, but the difference was not statistically significant at a 5% error level (*P* = 0.062). Gender, age, dmft value and annual household income did not have a significant association with children’s utilization of oral health services.

**Table 3:** Bivariate comparisons of oral health service utilization in the past 12 months (n = 1425)

Variables	Number of service users	Percentage (%)	*P value*
Predisposing factors			
Gender			0.731^a^
Male	331	45.0%	
Female	317	45.9%	
Age			0.498^a^
≤3 years old	224	44.3%	
> 3 years old	424	46.1%	
Parental oral health knowledge and attitude score			0.062^a^
< 5	284	42.8%	
≥ 5	364	47.8%	
Occupation of parent or caregiver			0.027^a^
Administrators/Professional	406	47.9%	
Other	242	41.9%	
Final education degree of the parent or caregiver			0.043^a^
High school or lower	195	42.4%	
University or college	263	44.5%	
Master’s degree or higher	190	50.8%	
Enabling factors			
Annual household income			0.260^a^
≤100,000 CNY	300	43.9%	
> 100,000 CNY	348	46.9%	
Kindergarten attendance with regular resource of oral health care			< 0.001^a^
Yes	492	49.5%	
No	156	36.2%	
Need factors			
Parental perceived oral health status of child			< 0.001^a^
Excellent/good	331	37.5%	
Fair	218	55.5%	
Poor	99	66.0%	
Dental pain in the past 12 months			< 0.001^a^
Yes	244	66.7%	
No	372	36.7%	
Unknown	32	43.2%	
Dmft value			0.030^b^

^a^*P* value calculated by Chi-square test

^b^*P* value calculated by t-test

Table 4 presents the final explanatory model of oral health service utilization in the past 12 months obtained from the multivariate logistic regression. Children who attended a kindergarten with regular oral health resources were more likely to utilize those services (OR = 2.38, CI = 1.81–3.12, *P* < 0.001). Children who had dental pain experiences tended to utilize oral health services (OR = 4.23, CI = 3.13–5.71, *P* < 0.001). Parents or caregivers who rated their children’s oral status as “poor” (OR = 2.23, CI = 1.48–3.35, *P* < 0.001), and parents who rated their child’s oral status as “fair” (OR = 1.63, CI = 1.25–2.12, *P* < 0.001) tended to take their children to a dentist compared to parents who rated their children’s oral health status as “excellent or good”. Children with higher dmft values were more likely to use oral health services (OR = 1.05, CI = 1.00–1.11, *P* = 0.039).

**Table 4:** Logistic regression model of oral health service utilization in the past 12 months (*n* = 1425)

Variables	*P* value	OR	95% CI
Predisposing factors			
Gender			
Male		1.00	1.00
Female	0.424	1.10	(0.87,1.37)
Age			
≤3 years old		1.00	1.00
> 3 years old	0.068	1.26	(0.98,1.61)
Parental oral health knowledge and attitude score			
< 5		1.00	1.00
≥ 5	0.055	1.25	(1.00,1.57)
Final education degree of the parent/caregiver	0.076^a^		
High school or lower		1.00	1.00
University or college	0.465^b^	1.11	(0.84,1.48)
Master’s degree or higher	0.104^b^	1.31	(0.95,1.82)
Occupation of parent or caregiver			
Administrator/professional		1.00	1.00
Other	0.186	0.85	(0.661.08)
Enabling factors			
Annual household income			
≤100,000 CNY		1.00	1.00
> 100,000 CNY	0.808	0.96	(0.70,1.32)
Kindergarten attendance with regular resource of oral health care			
No		1.00	1.00
Yes	< 0.001	2.38	(1.81,3.12)
Need factors			
Parental perceived oral health status of child	< 0.001^a^		
Excellent/good		1.00	1.00
Fair	< 0.001^b^	1.63	(1.25,2.12)
Poor	< 0.001^b^	2.23	(1.48,3.35)
Dental pain in the past 12 months	< 0.001^a^		
No		1.00	1.00
Yes	< 0.001^b^	4.23	(3.13,5.71)
Unknown	0.877^b^	1.04	(0.63,1.71)
Dmft value	0.049	1.05	(1.00,1.11)

^a^Overall P values for categorical variables (if more than 2 categories)

^b^Comparisons between the categories and the reference category (if more than 2 categories)

### Factors associated with the number of dental visits in the last 12 months

The results of the NBR are shown in Table 5. Children with more frequent dental visits in the past 12 months were more likely to be older than 3 years, have had their oral health status rated as “poor” or “fair”, have had regular resources for oral health, have had higher dmft values, have had dental pain experiences in the past 12 months, and have parents or caregivers who have a Master’s degree.

**Table 5:** Negative binomial regression model of oral health service utilization in the past 12 months (*n* = 1425)

Variables	*P* value	IRR (95% CI)
Predisposing factors		
Gender		
Male		1.00
Female	0.091	1.15 (0.98,1.36)
Age		
≤3 years old		1.00
> 3 years old	< 0.001	1.49 (1.24,1.79)
Parental oral health knowledge and attitude score		
< 5		1.00
≥ 5	0.060	1.17 (0.99,1.39)
Final education degree of the parent/caregiver	0.009^a^	
High school or lower		1.00
University or college	0.387^b^	1.09 (0.89,1.35)
Master’s degree or higher	0.012^b^	1.35 (1.07,1.71)
Occupation of parent or caregiver		
Administrator/Professional		1.00
Other	0.607	1.05 (0.87,1.26)
Annual household income		
≤100,000 CNY		1.00
> 100,000 CNY	0.619	0.94 (0.75,1.19)
Kindergarten attendance with regular source of oral health care		
No		1.00
Yes	< 0.001	2.23 (1.82,2.76)
Need factors		
Parental perceived oral health status of child	< 0.001^a^	
Excellent/good		1.00
Fair	0.041^b^	1.23 (1.01,1.49)
Poor	< 0.001^b^	2.09 (1.59,2.75)
Dental pain in the past 12 months	< 0.001^a^	
No		1.00
Yes	< 0.001^b^	2.05(1.67,2.52)
Unknown	0.501^b^	1.14(0.78,1.66)
Dmft value	0.021	1.04(1.01,1.08)

^a^Overall *P* values for categorical variables (if more than 2 categories)

^b^Comparisons between the categories and the reference category (if more than 2 categories)

## Discussion

Our study focused on the patterns of oral health service utilization and the related factors among preschool children in Beijing, with reference to the Andersen behavioral model. The prevalence of dental visits among preschool children in Beijing (45.5%) in our study was much higher than the prevalence among 5-year-old children nationwide in China (15.0%) and in Beijing (18.9%) [13], and it was higher than the prevalence in other regions of China (12.3%~ 42.3%) [3–8] but lower than the prevalence in some developed countries (52% to 84%) [16, 18, 23, 37]. We found that most preschool children in the study population were more likely to have dental visits when they experienced dental pain. In line with the findings of Hoeft et al. [10], children tended to receive routine checkups and dental preventive measures once they had an initial experience of visiting a dentist.

This study also confirms previous reports regarding the significant association of various factors with oral health service utilization among children based on the Andersen model, which includes predisposing factors (e.g., age [15, 20, 21, 23, 28] and education level of the parents or caregivers [18, 20, 23]), enabling factors (e.g., regular resources for oral health care [28, 29]) and need factors (e.g., children’s oral health status [24, 25, 28–30], dental pain experiences [19, 38], and dmft value [25, 39]).

Predisposing factors include factors related to children and factors related to parents or caregivers. The demographic factors of children, such as age and gender, are predisposing variables in the Andersen model, and they were found to be influencing factors of dental care utilization in children in some previous studies [19, 23, 25, 40]. The current study did not find a relationship between whether a child utilized dental care or not in the past 12 months and age or gender, but the number of dental visits was significantly associated with age, but not gender. One probable explanation is that oral health problems can increase as the children grow [19], and older children may have more teeth and more serious carious conditions if they do not receive proper treatment in time; thus, they need more dental visits. On the other hand, younger children may not be able to fully express unpleasant feelings related to tooth problems and are less likely to cooperate with dentists; thus, they receive simple treatment steps. For factors related to parents or caregivers, educational attainment was related to the number of dental visits. This connection might be because parents with a higher education level are better able to understand and critically follow treatment plans prescribed by dentists, and to use health-related information from social communications or from the Internet [41]. The occupation of parents or caregivers was a significant factor in bivariate analysis, though it was not significant in the logistic regression model when the education level was controlled. That result might have arisen because education level is usually closely associated with occupation. Although some studies found an association between oral health service utilization and the oral health beliefs of parents or caregivers [25–27], our study did not show a significant association. Hence, we suppose that in our study sample, oral health beliefs of parents or caregivers were less important when compared with the other determinants.

Access to oral health care resources is an important enabling factor in the Andersen model [31]. As in previous studies [24, 27, 28], our study indicated a strong association between access to regular oral health care resources and oral health service utilization among children. These results implied that kindergartens and primary schools are the prime public facilities in which to provide children with regular oral health care through governmental agencies or other health service providers. In this study, the annual household income, which is an enabling variable related to the family, was not associated in the bivariate and multivariate analysis, which agreed with the results of a study conducted in Brazil [24]. In this study, the absence of a statistical association between income and oral health service utilization could have occurred due to strong similarities in the socioeconomic status of the sample population. The influence of income on access to oral health service utilization tends to be more significant in developed countries where these services are predominantly private, which could also help explain our result [42].

One noticeable finding of this study was the significance of the need factors related to dental visits among the study population. Need factors, regardless of whether they are perceived or evaluated, are always direct reasons for oral health service utilization. The perception of a child’s oral health status by their parents or caregivers and children’s dental pain experiences were the strongest determinants of oral health service utilization in the present study. Children whose oral health status was rated as “fair or poor” may have more serious oral problems that may require immediate intervention, and unfavorable parental perceptions of their child’s oral health status could motivate them to seek dental care for their children [19]. Dental pain is the most common reason for dental visits in most developing counties [19, 38], and that was also observed in this study. Dental pain in children is mostly caused by caries and the dmft value is seen as the evaluated need for caries treatment in children. Our results also confirmed that of a previous study which found that dmft value was positively associated with oral health utilization [39].

Some limitations of this study need to be kept in mind when extrapolating the results to actual application. First, the current sampling methods were only for preschool children who attended kindergartens, whereas some vulnerable populations with lower socioeconomic status and with physical or mental disabilities do not go to kindergartens. Second, the final fitted models may not have included all variables associated with the utilization of oral health services in this population. For example, a child’s dental insurance coverage [40] and their parents’ own oral health experiences [2, 27] have been shown to be important predictors of a child’s use of oral health care services but were not collected in the questionnaire used in our study. However, most known determinants were included, and the model could still be explanatory. As the planned next stage of our study, national survey data will be used if possible (the results of the newest one, namely, the 4th National Oral Health Survey in China, will be released in 2018), and more comprehensive variables will be included into the model then to better identify the determinants of oral health service utilization among the whole younger population in China. Third, our study utilizing Andersen’s model has examined individual components (e.g., predisposing factors, enabling factors and need factors) and their relationships with oral health service utilization, while how these interact in influencing the use of dental services was not fully tested [43].

Our findings have some implications for policies to increase oral health service utilization in children. First, access to oral health service resources should be brought to the attention of the government and health service providers. Second, the importance of early dental visits and regular check-ups should also be emphasized through different methods of oral health education not only for parents and caregivers but also for children to create a checkup-oriented or prevention-oriented utilization pattern instead of a problem-oriented pattern among preschool children. For instance, mass media campaigns and oral health education programs based on community and kindergartens could be excellent choices.

## Conclusion

In conclusion, we found a strong association between the access to regular oral health care resources and oral health service utilization among preschool children. Children whose oral health status as perceived by parents/caregivers was worse and who had more dental pain experiences were more likely to have utilized oral health services in China.

## Additional files

Additional file 1: Oral health questionnaire for parents/caregivers. (DOCX 18 kb)

Additional file 2: Oral health assessment form for children. (DOC 69 kb)

## Abbreviations

CI: Confidence interval
CPI: Community Periodontal Index
dmft: Decayed, missing and filled teeth in primary dentition
ECC: Early childhood caries
IRR: Incidence rate ratio
LR: Likelihood ratio test of alpha = 0
NBR: Negative binomial regression
OR: Odd ratio
PEN: Predisposing factor, enabling factor and need factor
VIF: Variance inflation factor
WHO: World Health Organization
