# Supplementary material for: Oral health service utilization patterns among preschool children in Beijing, China
Source: BMC Oral Health. 2018 Mar 6;18:31. doi: 10.1186/s12903-018-0494-6 (PMC5838987; doi:10.1186/s12903-018-0494-6)
Supplement: Supplementary file 1 — Oral health questionnaire for parents/caregivers. (DOCX 18 kb) [file 12903_2018_494_MOESM1_ESM.docx]

**Oral health questionnaire for parents/caregivers**

*This study is conducted by Peking University School and Hospital of Stomatology. The following questionnaire is very helpful for us to provide your child with more appropriate oral health care plan. We hope you can read the following questions and answer every item carefully. Thank you very much for your cooperation.*

Code:

Kindergarten: Class: Child’s name:

Child’s gender: Child’s birth date:

1.What is your relationship with the child? **(Only one choice is available)**

1) father 2) mother 3) grandparent

4) relatives 5) babysitter

2. Who are your child taken care of mostly in daily life? **(Only one choice is available)**

1) father 2) mother 3) grandparent

4) relatives 5) babysitter

3. What is the feeding habit in 4 months from your child’s birth? **(Only one choice is available)**

1) only breast feeding 2) mostly breast feeding

3) only artificial feeding 4) mostly artificial feeding

4) half breast feeding and half artificial feeding

4. How often do your child eat/drink the following foods/beverages? **(Only one choice is available for every sub-question)**

a) carbonate beverages (e.g., cola and spirit)

1). more than twice a day 2). once a day 3). less than once a day

b) desserts (e.g., biscuits, cake and bread)

1). more than twice a day 2). once a day 3). less than once a day

c) candies /chocolates

1). more than twice a day 2). once a day 3). less than once a day

5.Do your child eat desserts or drink sweet beverages before sleep? **(Only one choice is available)**

1) often 2) seldom

3) never (if you choose this answer then turn to question No.7 directly)

6. Do your child brush teeth after eating desserts or drinking sweet beverages? **(Only one choice is available)**

1) often 2) seldom 3) never

7. When do your child start brush teeth? **(Only one choice is available)**

1) 6 months after birth 2) 1 year old 3) 2 years old

4) 3 years old 5) never or seldom brush teeth

8. As for your child’s teeth brushing, **(Only one choice is available for every sub-question)**

a) how often do you help your child brush his/her teeth in the latest month?

1) never 2) seldom 3) sometimes 4) every week 5) every day

b) how often do you check after your child brush his/her teeth in the latest month?

1) never 2) seldom 3) sometimes 4) every week 5) every day

9. How often did your child have dental pain experiences in the past 12 months? **(Only one choice is available)**

1) never 2) sometimes 3) often 4) unknown

10. When was the last dental visit from now? **(Only one choice is available)**

1) never visited dentist 2) more than 2 years 3) 1-2 years

3) 6-12 months 4) less than 6 months

(Caution: If you choose the answer 1), 2), 3), please answer the question No.14 directly)

11.In the past 12 months, how many times have your child visited dentists?

Please write down a number here: _____

12. What was the reason for your child’s last dental visit? **(Only one choice is available)**

1) emergency for trauma 2) acute toothache 3) chronic toothache

4) examinations for oral problems 5) routine check-up

6) application of preventive measures 7) aesthetic needs

13. How much did you spend on your child’s dental visit (expenditures on medication and treatment)? Please write down here: _____yuan

14. a) How do you evaluate the oral health status of your child? **(Only one choice is available for every sub-question)**

1)excellent 2) good 3) fair 4) poor 5) very poor

b) How do you evaluate the general health status of your child?

1)excellent 2) good 3) fair 4) poor 5) very poor

15. How do you think of the following points of view? **(Only one choice is available for every sub-question)**

a) Our teeth are instinctive and there is little to do with self-protection.

1) agree 2) disagree 8) do not care

b) Oral health is very important to our life.

1) agree 2) disagree 8) do not care

c) Poor conditions of the mother’s dental health would affect the children’s dental health condition.

1) agree 2) disagree 8) do not care

d) There is no need to treat the primary teeth when decayed.

1) agree 2) disagree 8) do not care

16. How do you think of the following statements? **(Only one choice is available for every sub-question)**

a) It is not clarified that fluoride application could prevent dental caries.

1) correct 2) incorrect 8) sorry, I do not know

b) Pit and fissure sealants can protect children from dental caries.

1) correct 2) incorrect 8) sorry, I do not know

c) Brushing teeth twice a day can protect children from dental caries.

1) correct 2) incorrect 8) sorry, I do not know

d) Routine oral health check-ups could help to find out oral health problems in time.

1) correct 2) incorrect 8) sorry, I do not know

17. What is your highest educational degree? **(Only one choice is available)**

1) illiteracy 2) primary school 3) junior high school

4) senior high school 5) technical secondary school 6) junior college

7) bachelor’s degree 8) master’s degree and above

18. What is your occupation? **(Only one choice is available)**

1) administrator 2) professional 3) office clerk

4) business or service industry staff 5) agricultural staff

6) transportation staff 7) soldier 8) others 9) unemployment

19. Total household income of your family in the past 1 year is: **(Only one choice is available)**

1) <10,000 CNY 2) 10,000-30,000 CNY 3) 30,000-50,000 CNY

4) 50,000-100,000 CNY 5) 100,000-300,000 CNY 6) 300,000-500,000 CNY

7) >500,000 CNY

**Thank you again for your cooperation!**
