# Supplementary material for: Oral health service utilization patterns among preschool children in Beijing, China
Source: BMC Oral Health. 2018 Mar 6;18:31. doi: 10.1186/s12903-018-0494-6 (PMC5838987; doi:10.1186/s12903-018-0494-6)
Supplement: Supplementary file 2 — Oral health assessment form for children. (DOC 69 kb) [file 12903_2018_494_MOESM2_ESM.doc]

**Oral Health Assessment Form for Children**

**General information**

**Kindergarten: _______________ Class: ____________ Name: ________________**

**Gender: ____________ Birthdate: _________**

**Oral health status**

**1．**Dentition status by tooth surface

| **Mes-M** |  |  |  |  |  |  |  |  |  |  |  |  | **Mes-M** |
| --- | --- | --- | --- | --- | --- | --- | --- | --- | --- | --- | --- | --- | --- |
| **Dis-D** |  |  |  |  |  |  |  |  |  |  |  |  | **Dis-D** |
| **Buc-B** |  |  |  |  |  |  |  |  |  |  |  |  | **Buc-B** |
| **Lin-L** |  |  |  |  |  |  |  |  |  |  |  |  | **Lin-L** |
| **Occ-O** |  |  |  |  | | | | | |  |  |  | **Occ-O** |
|  | **16** | **15** | **14** | **13** | **12** | **11** | **21** | **22** | **23** | **24** | **25** | **26** |  |
|  |  | **55** | **54** | **53** | **52** | **51** | **61** | **62** | **63** | **64** | **65** |  |  |
|  |  | **85** | **84** | **83** | **82** | **81** | **71** | **72** | **73** | **74** | **75** |  |  |
|  | **46** | **45** | **44** | **43** | **42** | **41** | **31** | **32** | **33** | **34** | **35** | **36** |  |
| **Occ-O** |  |  |  |  | | | | | |  |  |  | **Occ-O** |
| **Lin-L** |  |  |  |  |  |  |  |  |  |  |  |  | **Lin-L** |
| **Buc-B** |  |  |  |  |  |  |  |  |  |  |  |  | **Buc-B** |
| **Dis-D** |  |  |  |  |  |  |  |  |  |  |  |  | **Dis-D** |
| **Mes-M** |  |  |  |  |  |  |  |  |  |  |  |  | **Mes-M** |

**2．Periodontal status（DI-S）**

| **DI-S** |  |  |  |  | **DI-S** |
| --- | --- | --- | --- | --- | --- |
|  | **55（B）** | **51/11（La）** |  | **65（B）** |  |
|  | **85（L）** |  | **71/31（La）** | **75（L）** |  |
| **DI-S** |  |  |  |  | **DI-S** |

Examiner ____________ Date _________

**Criterion for Oral Health Assessment**

**1．** Dentition status by tooth surface

| **Primary teeth** | **status** |  | **Permanent teeth** | **status** |
| --- | --- | --- | --- | --- |
| 0 | Sound |  | A | Sound |
| 1 | Caries |  | B | Caries |
| 2 | Filled with caries |  | C | Filled with caries |
| 3 | Filled, no caries |  | D | Filled, no caries |
| 4 | Missing due to caries |  | E | Missing due to caries |
| 5 | Missing for another reason |  | F | Missing for another reason |
| 6 | Fissure sealant |  | S | Fissure sealant |
| 7 | Fix prosthesis/crown. abutment, veneer |  | G | Fix prosthesis/crown. abutment, veneer |
| T | Trauma |  | T | Trauma |
| X | Not record |  | X | Not record |
|  |  |  | 8 | Not erupt |

**2．Periodontal status (DI-S)**

| **Code** | **status** |
| --- | --- |
| 0 | No debris seen on the facial surface of the tooth |
| 1 | Debris covered less than 1/3 of the facial surface of the tooth |
| 2 | Debris covered about 1/3-2/3 of the facial surface of the tooth |
| 3 | Debris covered more than 2/3 of the facial surface of the tooth |
| 9 | Not record |
